# Sex‐specific characterization of aortic function and inflammation in a new diet‐induced mouse model of metabolic syndrome

**DOI:** 10.1096/fj.202401871R

**Published:** 2025-03-04

**Authors:** Vivian Tran, Holly Brettle, Henry Diep, Hericka Bruna Figueiredo Galvao, Kerry V. Fanson, Christopher G. Sobey, Grant R. Drummond, Antony Vinh, Maria Jelinic

**Affiliations:** ^1^ Department of Microbiology, Anatomy Physiology and Pharmacology, Centre for Cardiovascular Biology and Disease Research, La Trobe Institute for Molecular Science, School of Agriculture, Biomedicine and Environment La Trobe University Bundoora Victoria Australia; ^2^ Department of Animal, Plant and Soil Sciences, School of Agriculture, Biomedicine and Environment La Trobe University Bundoora Victoria Australia

## Abstract

Perivascular adipose tissue (PVAT) expansion promotes inflammation and vascular dysfunction in metabolic syndrome (MetS), but the sexual dimorphisms of PVAT are poorly understood. Using a new mouse model of diet‐induced MetS, we characterized the aorta and determined the influence of PVAT on vascular function in males and females. Six‐week‐old C57BL/6 mice were fed either a high‐fat diet (43% kcal in food) with high sugar and salt in their drinking water (10% high fructose corn syrup and 0.9% NaCl; HFSS), or a normal chow diet (NCD) for 10 weeks. The aorta was characterized at endpoint using pin myography, flow cytometry, bulk RNA‐sequencing, GSEA analysis, and histology. Compared to NCD‐fed mice, HFSS‐fed mice displayed higher weight gain, fasting blood glucose, systolic blood pressure, aortic fibrosis, and perivascular adipocyte cross‐sectional area, regardless of sex (*p* < .05). Circulating adiponectin levels were also higher in HFSS‐fed males compared to NCD males. PVAT enhanced U46619‐mediated contraction in HFSS males only. HFSS increased the expression of immune regulation genes in female PVAT and ion transport genes in male PVAT but had no effect on total numbers of immune cells in the aorta in either sex. Despite having similar effects on metabolic parameters in males and females, HFSS caused contrasting effects on vascular function with and without PVAT. These data highlight the sexual dimorphisms of PVAT in regulating the vasculature in healthy and diseased states.

## Graphical abstract

C57BL/6 mice were fed either a high‐fat diet with high sugar and salt in their drinking water (HFSS) or a normal chow diet (NCD) for 10 weeks. Despite having similar effects on metabolic parameters, aortic fibrosis, and perivascular adipocyte size in both sexes, HFSS caused contrasting effects on circulating adiponectin levels, PVAT gene expression, and aortic function.

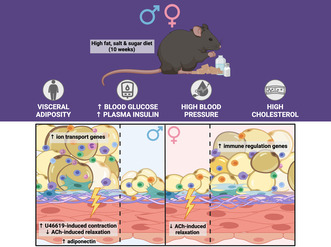

## 1 INTRODUCTION

Cardiovascular disease is the leading cause of global mortality and is estimated to contribute to 17.8 million annual deaths worldwide.^1^, ^2^ MetS is a clinical term used to describe a cluster of metabolic abnormalities specifically —, three or more of the following: abdominal obesity, hyperglycemia, insulin resistance, high blood pressure, and dyslipidemia, all of which are recognized as risk factors for cardiovascular disease.^3^

Animal models are important tools for improving our understanding of MetS disease progression and identifying/testing potential new therapeutic approaches. Diet modifications are often used to induce MetS in rodents.^4^ These diet modifications include excessive consumption of carbohydrates and/or fats, which can induce obesity, hyperglycemia, and endothelial dysfunction after 10 weeks.^5^, ^6^ While high‐fat diet rodent models usually display most of the features of MetS, patients with MetS often consume a higher proportion of simple carbohydrates than what is seen in the high‐fat diet models described in the literature.^7^ Diets that are both high in fat and carbohydrates also elicit the desired features of MetS in rodents and mimic a typical poor Western diet of a MetS patient. Therefore, these models are more clinically relevant than those using diets only high in fat.^7^

One major limitation of diet‐induced mouse models of MetS is that many studies exclude female mice as they are more resistant to developing diet‐induced MetS.^8^ Clinically, 83% of men and 86% of women are diagnosed with MetS; therefore, excluding females in preclinical studies neglects a significant portion of the clinical population.^9^ Moreover, sex‐specific pathways in the pathophysiology of MetS emphasize the importance of studying both sexes for future advances in developing therapies that are suited to all patients with MetS.^10^

The presentation of MetS differs between sexes. In premenopausal women, abdominal obesity is the most common MetS feature with increased triglycerides, low high‐density lipoproteins (HDL) and increased waist circumference.^11^ Men, however, exhibit increased triglycerides, low HDL, and hypertension.^11^ Sex‐related factors associated with biological traits and functional features change noticeably with age, especially during and after menopause, where these differences are abolished.^12^ In males with MetS, there is a greater accumulation of visceral/abdominal adipose tissue, which is associated with the development of cardiovascular diseases, insulin resistance, and type 2 diabetes.^13^, ^14^, ^15^ Conversely, females with MetS accumulate adipose tissue in the gluteal‐femoral depots, which has ‘disease‐protective’ properties.^13^, ^14^, ^15^ Sex is also a major biological variable in inflammatory responses. Indeed, in patients with MetS, there are striking differences in proinflammatory profiles between sexes. However, no studies to date have characterized the aortic leukocyte population in both sexes in the setting of MetS. Studies in other white adipose beds have shown increased CD8^+^ effector T cell infiltration in gonadal white adipose tissues in obese high‐fat diet‐fed males. In contrast, anti‐inflammatory Treg cells are increased in the gonadal adipose tissue of obese high‐fat diet‐fed females. This anti‐inflammatory phenotype likely contributes to the protection from the metabolic abnormalities associated with a high‐fat diet observed in obese females.^16^, ^17^, ^18^, ^19^, ^20^, ^21^, ^22^

Vascular dysfunction is a major driver of disease progression and end organ damage in MetS. However, it is unclear whether there are sex‐specific effects of MetS on vascular function. Perivascular adipose tissue (PVAT) surrounds blood vessels and is a recognized regulator of vascular function. Healthy PVAT typically releases a wide variety of adipokines and vasodilatory mediators to facilitate cellular communication and modulate vascular tone.^23^, ^24^ In hypertension and obesity, perivascular adipocytes undergo hypertrophy and hyperplasia, with impaired angiogenesis.^25^ Dysfunction of the PVAT ensues, leading to a shift toward the production of proinflammatory cytokines and recruitment of immune cells into the PVAT.^26^ This chronic low‐grade inflammatory state further promotes oxidative stress and dysregulates local adipokine release, impairing vascular function.^23^ There are discrepancies in the literature regarding the effects of MetS on PVAT and vascular function. This is largely due to a variety of approaches used to model MetS and differences in the vascular beds studied.^27^, ^28^, ^29^ Moreover, most studies to date focus on males, largely neglecting the characterization of PVAT in females with MetS.^30^ Therefore, we aimed to use a new, robust, clinically relevant mouse model of MetS to characterize the sex‐specific responses to a high‐fat, salt, and sugar diet (HFSS) and its effects on aortic inflammation and function. Our study highlights the greater susceptibility of males to MetS and the associated vascular dysfunction, which is likely driven by the detrimental nature of PVAT in males with MetS.

## 2 MATERIALS AND METHODS

### 2.1 Animal model of diet‐induced metabolic syndrome

Male and female C57BL/6J mice were obtained from the La Trobe Animal Research and Teaching Facility and housed in individually ventilated cages with access to food and water ad libitum. To induce MetS, mice aged 5–7 weeks were randomly assigned to either a normal chow diet (NCD; 20% total crude protein, 5% crude fat, 6% crude fiber, 0.5% added salt, 0.8% calcium and 0.45% phosphorus; Barastoc WEHI mouse breeder cubes, irradiated; Ridley, Australia) or a high fat, salt, and sugar diet (HFSS; 23% total crude protein, 23.5% total fat, 5.4% crude fiber; SF04‐001, Specialty Feeds Perth, Western Australia; 0.9% sodium chloride; Baxter Professional, Illinois, United States of America; 10% glucose and fructose; Chem‐supply, Adelaide, Australia in the drinking water) for 10 weeks. All animal experimentation complied with the National Health and Medical Research Council (NHMRC) of Australia Code of Practice for the care and use of animals for scientific purposes and ARRIVE guidelines. Animal care and experimental protocols were approved by the La Trobe Animal Ethics committee (AEC19009).

### 2.2 Blood pressure measurement

Systolic BP (SBP) was measured weekly by a non‐invasive tail cuff plethysmography (MC4000 Multi Channel Blood Pressure Analysis System, Hatteras Instruments, Cary, NC, USA) as previously described.^31^ Mice were acclimated 1 week prior to BP measurements to minimize stress. Mice underwent 10 acclimation cycles, followed by 4 sets of 15 cycles. The data output was recorded using MC4000 Blood Pressure Analysis System software. Mean SBP measurements were recorded weekly during the 10‐week HFSS regimen.

### 2.3 Fasted blood collection

Fasted bloods were collected fortnightly. Mice were fasted for 6 h (from 8:00 AM) with free access to water. Blood glucose was measured in samples from the saphenous vein using a handheld Accu‐Chek® Guide glucose meter per manufacturer instructions (NSW, Australia). Blood was collected (~120 μL) for plasma isolation to measure insulin using a Mouse Ultrasensitive Insulin ELISA kit (ALPCO Diagnostics, Salem, NH, USA) and Mouse Adiponectin Quantikine ELISA kit (R&D Systems, Minneapolis, MN, USA) as per manufacturer instructions.^32^

### 2.4 Measurement of aortic stiffness

At endpoint, Pulse‐wave Doppler images and EKV™ retrospective acquired B‐Mode images of the suprarenal region of the abdominal aortae were obtained using a MS‐400 ultrasound transducer (Vevo 2100; FujiFilm VisualSonics Inc., Canada). Data were analyzed using the VevoLab and VevoVasc software (Supp Figure 1; FujiFilm VisualSonics Inc., Canada) and the diameter‐velocity loop method was used to obtain measures of aortic pulse wave velocity (PWV).^33^

**FIGURE 1:**
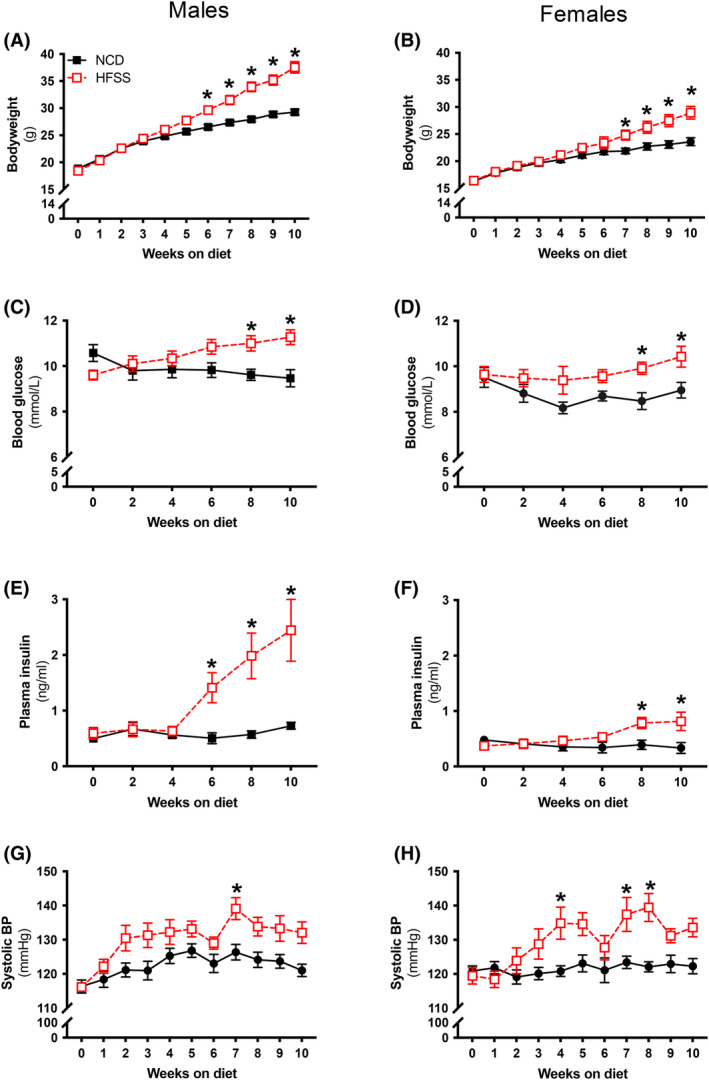
High‐fat, salt, and sugar diet‐induced metabolic disturbances in both sexes. Body weight (A and B), fasting blood glucose (C and D), fasting plasma insulin (E and F) and systolic blood pressure (G and H) from C57BL/6 male and female mice fed a normal chow diet (NCD; black; ●) or high‐fat, salt and sugar diet (HFSS; red; □). Values are expressed as mean ± SEM. *p* < .05 for 2‐way ANOVA with Sidak's multiple comparison and Mixed‐effect analysis. *NCD vs. HFSS, A–D *n* = 9–16 per group, E and F *n* = 6–7 per group, G and H *n* = 9–16 per group.

### 2.5 Plasma steroid measurements

Plasma steroids were extracted twice with diethyl ether to remove potential interfering substances.^34^ The supernatant, containing extracted steroids, was collected, dried, and reconstituted in phosphate buffer. All hormones were quantified using commercial ELISAs (Arbor Assays, Ann Arbor, Michigan). Plasma progesterone (product number ISWE003) was quantified in both sexes. Due to limited sample volumes, testosterone (ISWE001) was only quantified in males and estradiol (ISWE0008) was only quantified in females. Briefly, a 96‐well flat‐bottomed microtiter plate was coated with *donkey anti‐sheep immunoglobulin*. The plate was washed. Samples, standards (10–0.078 ng/mL) and controls were loaded into each well, followed by the corresponding enzyme‐labeled antibody. The plate was incubated for 2 h, then washed. 3,3′,5,5’‐Tetramethylbenzidine substrate solution was added to each well. After 30 min, the reaction was stopped using 0.5 M H_2_SO_4_, and the plate was read at 450 nm and 620 nm using the SPECTROstar Nano Plate reader (BMG Labtech, Mornington Victoria, Australia). All samples were analyzed in duplicate. To assess matrix interference and determine the dilution factor for each assay, a parallelism between the standard curve and a serially diluted sample pool was performed for all samples.

### 2.6 HbA1c and lipid measurements

Mice were euthanized using carbon dioxide asphyxiation followed by cardiac puncture and injection of 0.1 mL of enoxaparin sodium (400 units; Clexane®, Auckland, NZ). Blood samples were measured for glycated hemoglobin (HbA1c), total cholesterol, and triglycerides using the Roche Cobas b 101 Monitoring System (Basel, Switzerland^32^). The remaining blood samples were placed on ice for flow cytometric analysis, after which mice were intracardially perfused with phosphate‐buffered saline (PBS).

### 2.7 Isolation of aortae

Following perfusion, aortae were harvested and placed in ice‐cold PBS for either flow cytometry, histology, or bulk RNA sequencing analyses. Lymph nodes and excess visceral tissues were carefully removed via microdissection.^35^ For pin myography studies, aortae were dissected in ice‐cold Krebs bicarbonate solution (mmol/L; NaCl 2973.1, KCl 117.4, MgSO_4_.7H_2_O 29.3, KH_2_PO_4_ 29.6).

### 2.8 Pin myography

Vascular function of the abdominal aorta with and without PVAT was assessed as described previously.^36^ Aortic rings (2 mm in length) were mounted on a 4‐channel Mulvany‐Halpern Myograph (model 610 M, Danish Myo Technology, Aarhus, Denmark) and allowed to stabilize at zero tension for 15 min, followed by normalization and equilibration.^37^ Starting from an unloaded state defined as the maximum diameter with zero force, the circumferential length was then slowly increased in a stepwise manner by manual adjustment. After a stable level of force, aortic rings were maximally contracted and activated using a high K^+^ solution (KPSS). All experiments were performed in Krebs bicarbonate solution (mmol/L; NaCl 2973.1, KCl 117.4, MgSO_4_.7H_2_O 29.3, KH_2_PO_4_ 29.6) at 37°C and bubbled with carbogen (95% O_2_ and 5% CO_2_). Changes in isometric tension were recorded using a Powerlab/Lab Chart data acquisition system (AD Instruments, Bella Vista, NSW, Australia). Vessels were maximally contracted with the thromboxane A_2_ mimetic U46619 (1 μM bolus). This was followed by the determination of endothelial integrity as described previously.^38^, ^39^ To assess vasorelaxant function, aortic rings with and without PVAT were pre‐contracted to 70–80% of maximum U46619 contraction using U46619, and cumulative concentration‐response curves to the endothelium‐dependent agonist acetylcholine (ACh; 0.1 nM – 10 mM) or the endothelium‐independent vasorelaxant NO donor sodium nitroprusside (SNP; 0.1 nM – 10 mM) were performed, as previously described.^37^ Additionally, separate aortic rings were used to test the contribution of NO synthase and cyclooxygenase to vasorelaxation responses to ACh. Vessels were pre‐incubated in the presence of a NO synthase inhibitor, Nω‐nitro‐ι‐arginine methyl ester (L‐NAME; 200 mM) or indomethacin (1 μM), a COX inhibitor, for 30 min prior to U46619 pre‐constriction and ACh vasorelaxation curves. Concentration‐response curves to the thromboxane mimetic U46619 were performed to test U46619‐mediated contraction.

### 2.9 Flow cytometry

Flow cytometry was performed on cell suspensions derived from freshly isolated aortae (including PVAT) as previously described.^35^ Aortae were minced with scissors and enzymatically digested in PBS containing MgCl_2_ and CaCl_2_ comprising a mixture of type XI collagenase (125 U/mL, C7657‐100MG), hyaluronidase (600 U/mL, H3884‐50MG) and type I‐S collagenase (450 U/mL, C1639‐50MG; all enzymes purchased from Sigma‐Aldrich, USA) for 1 h at 37°C, as previously described.^35^ Samples were then passed through a sterile 70 μm filter (BD Biosciences, USA) and cells were pelleted by centrifugation at 1500 × *g* RPM for 5 min at 4°C. Aortic cell suspensions were stained with an Aqua Live/dead cell viability stain (Invitrogen, USA; 1 in 1000 dilution in PBS) for 15 min at room temperature, followed by a master mix containing a cocktail of antibodies in MACS buffer (0.5% bovine serum albumin and 2 mM EDTA in PBS; full list of antibodies in Table S1). Lastly, stained cells were fixed in MACS buffer containing 1% formalin. All samples were analyzed using a CytoFLEX S analyzer (Beckman Coulter, Indianapolis, IN, USA) using CytExpert software version 2.5 (Beckman Coulter, USA). Immune cell populations were quantified using FlowJo software (BD Biosciences, version 10.8.1; gating strategy in Figure S2). Cell numbers were expressed as total cells per aorta.

### 2.10 Bulk RNA‐sequencing

Frozen aortic samples were sonicated in TRIzol™ (Life Technologies, USA), mixed with chloroform, and centrifuged at 824 rcf for 15 min at 4°C. The aqueous phase was collected, and RNA was extracted using the RNeasy® Micro Kit (Qiagen, USA). RNA was quantified using the NanoDrop One spectrophotometer (Thermo Scientific, USA). RNA was then stored at −80°C and sent to NovogeneAIT Genomics (Singapore) for cDNA library preparation and RNA sequencing. mRNA was purified from total RNA using poly‐T oligo‐attached magnetics and converted to cDNA, then purified using AMPure XP Beads (Beckman Coulter Life Sciences, USA). cDNA libraries were acquired by PCR amplification, and high‐throughput sequencing was conducted using the HiSeqTM2500 platform (Illumina, USA).

The results were mapped to the Ensembl‐released mouse genome sequence GRCm38 and annotation. Differential gene expression analysis was conducted using the DESeq R Package V.1.10.1, and *P*‐values were adjusted using the Benjamini‐Hochbergs approach for controlling the false discovery rate. Genes with an adjusted *p* < .05 were considered significantly differentially expressed.

Gene set enrichment analysis (GSEA) identifies incremental changes in gene expression of a group of genes within a gene set. This analysis was used to calculate enrichment scores of gene ontology terms. The Seurat (v4.1.1) “FindMarkers” function was used to calculate the relative expression of all sequenced genes with the minimum log2‐fold change, minimum percentage expression, and minimum percentage difference parameters set to “‐Inf” (negative infinity). Expressed genes were ranked from highest to lowest log2‐fold change. The clusterProfiler (v4.4.4)^40^, ^41^ “GSEA” function had a minimum and maximum gene set size of 40 and 500 genes, respectively. The Benjamini‐Hochberg correction was used to obtain gene ontology terms from the msigdbr package (v7.5.1)^42^, ^43^ with adjusted *p* < .01. Results were then visualized using the “gseaplot2” function from the enrichplot package (v1.18.3).

### 2.11 Reagents

Drugs for pin myography were purchased from Sigma‐Aldrich (St Louis, MO, USA), except for U46619 (Cayman Chemical, Ann Arbor, MI, USA). All drugs were dissolved in distilled water, except for indomethacin, which was dissolved in 0.1 mol/L sodium carbonate, and U46619, which was dissolved in 100% ethanol to create a stock solution at 1 mmol/L. All antibodies for flow cytometry were purchased from BioLegend, USA.

### 2.12 Histopathology

Aortic sections were fixed in 10% neutral buffered formalin, embedded in paraffin wax, and cut into 4 μm sections. Vessels were stained with Masson's trichrome and Verhoeff‐Van Gieson for histological analysis of adipocytes, aortic diameter, collagen, and elastin content. Each whole slide was scanned using the Aperio Scanscope AT Turbo, and raw virtual slide files were viewed using Aperio ImageScope software (Leica Biosystems Microsystems, Mount Waverley, VIC, Australia) to extract the whole image. Adipocyte cross‐sectional area was quantified in ImageJ (version 1.53 t, National Institute of Health, USA).

### 2.13 Statistical analysis

Results were expressed as mean ± SEM. All data analysis was performed in GraphPad Prism (version 7.0, GraphPad Software, San Diego, CA, USA). Data were analyzed using either mixed‐effects analysis or 2‐way repeated measures ANOVA where appropriate. Post hoc analyses were performed from ANOVA using Tukey's multiple comparison test or Sidak's multiple comparison test for mixed‐effects analysis. *p* < .05 was considered statistically significant.

## 3 RESULTS

### 3.1 HFSS induces MetS in both sexes through increased macronutrient intake

HFSS increased body weight in males and females compared to NCD (Figure 1A,B). Post hoc analyses revealed that increased body weight in HFSS mice started from week 6 in males (*p* < .05; Figure 1A), and from week 7 in females (*p* < .05; Figure 1B). HFSS‐induced hyperglycemia by week 8 on the diet for both sexes (*p* < .05; Figure 1C,D). HFSS increased fasting plasma insulin from week 6 of the diet regime for males (*p* < .05; Figure 1E) and week 8 of the diet regime for females (*p* < .05; Figure 1F). SBP was transiently increased in males at week 7 (*p =* .01; Figure 1G) on the diet, and weeks 4, 7, and 8 in females (*p* < .05; Figure 1H). Blood lipids were measured at endpoint to determine whether the HFSS diet induced dyslipidemia. HFSS increased blood cholesterol in males (*p* < .0001) and females (*p* < .0001; (Table 1)). HFSS increased blood triglycerides and lowered HbA1c in females only (*p* = .03; Table 1) HbA1c was not affected in males when compared to NCD control mice (Table 1). Macronutrient intake was measured during the diet period. Fat, carbohydrate, and salt intake were increased in males (fat: *p* = .0004; carbohydrate: *p* = .004; salt: *p* = .001; Table 2) and females (fat: *p* = .0004; carbohydrate: *p* = .003; salt: *p* = .002; Table 2) on an HFSS diet. Protein intake remained unchanged in all groups (Table 2). Overall, HFSS induced MetS in both male and female mice.

**TABLE 1:** Blood cholesterol, triglycerides, and glycated hemoglobin in normal control diet (NCD) and high fat, salt, and sugar (HFSS) diet‐fed male and female mice at study endpoint.

	Cholesterol (mmol/L)	Triglycerides (mmol/L)	HbA1c (%)
Males			
NCD	6.9 ± 0.17	10.4 ± 0.55	4.37 ± 0.08
HFSS	9.9 ± 0.36*	10.8 ± 0.45	4.51 ± 0.06
Females			
NCD	5.36 ± 0.14	7.9 ± 0.34	5.18 ± 0.31
HFSS	7.35 ± 0.17*	9.22 ± 0.45*	4.47 ± 0.10*

*Note*: Values are expressed as mean ± SEM, *p < .05 vs. NCD, 2‐way ANOVA with Tukey's multiple comparisons test.

**TABLE 2:** Average weekly macronutrient and salt intake in normal control diet (NCD) and high fat, salt, and sugar (HFSS) diet‐fed male and female mice.

	Fat (g)	Carbohydrate (g)	Protein (g)	Salt (g)
Males				
NCD	0.30 ± 0.04	0.35 ± 0.05	1.18 ± 0.17	0.03 ± 0.04
HFSS	1.56 ± 0.34*	0.71 ± 0.16*	1.50 ± 0.33	0.09 ± 0.01*
Females				
NCD	0.25 ± 0.03	0.30 ± 0.04	1.01 ± 0.14	0.03 ± 0.003
HFSS	1.49 ± 0.43*	0.68 ± 0.20*	1.43 ± 0.41	0.07 ± 0.005*

*Note*: Values are expressed as mean ± SEM, *p < .05 vs. NCD, 2‐way ANOVA with Tukey's multiple comparisons test.

### 3.2 HFSS increased aortic collagen and adipocyte size in both sexes

**FIGURE 2:**
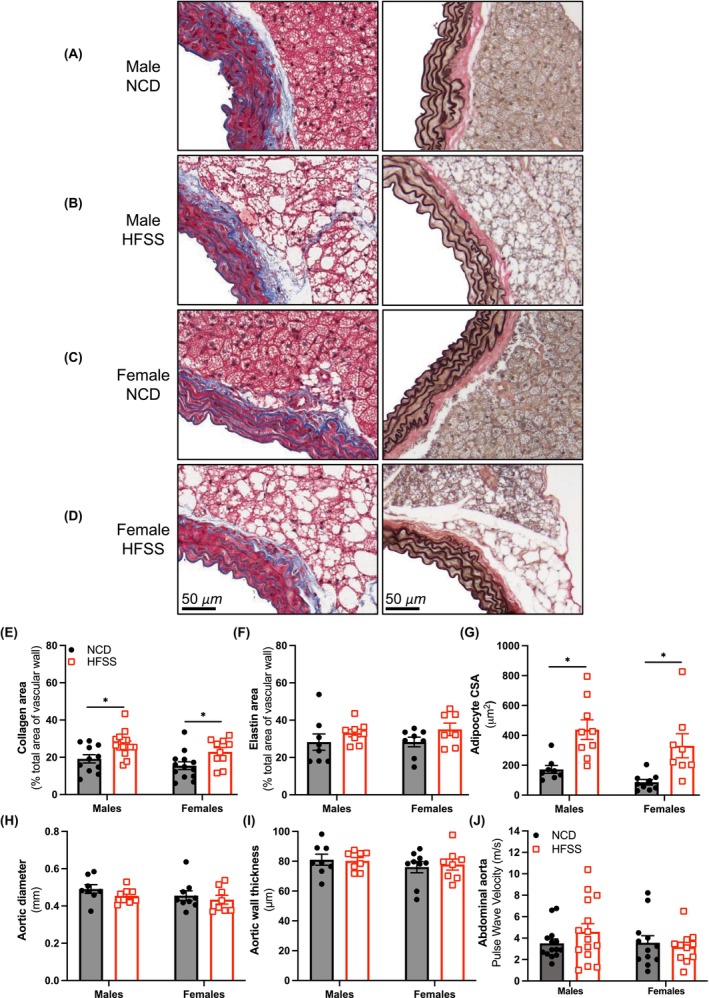
High‐fat, salt, and sugar increased the percentage of collagen in the aorta and aortic perivascular adipocyte size in male and female mice. Circulating adiponectin increased over time in high‐fat, salt, and sugar‐fed males. Representative images of the aorta stained with Masson's trichrome from (A) NCD male, (B) HFSS male, (C) NCD female and (D) HFSS female. Images were scanned at 10x magnification. Collagen area (as percentage of total area of vascular wall; E), elastin area (as percentage of total area of vascular wall; F), average adipocyte cross sectional area (CSA; G), aortic diameter (H), aortic wall thickness (μm ;I) and pulse wave velocity (of abdominal aorta m/s; J) in C57BL/6 male and female mice fed a NCD (●) or HFSS diet (□). Scale bar = 100 μm. Values are expressed as mean ± SEM, *n* = 8–15 per group. *p < .05 vs. NCD, for Unpaired *t*‐test and 2‐way ANOVA with Sidak's multiple comparison.

Masson's trichrome was used to assess the effect of HFSS on aortic collagen and elastin content and perivascular adipocyte size (Figure 2). Aortic collagen content (males: *p* = .008; females: *p* = .02; Figure 2A) was increased in response to HFSS in both sexes, and aortic elastin content remained unchanged (Figure 2B). Perivascular cross‐sectional area (males: *p* = .03; females: *p* = .007; Figure 2C) was increased in response to HFSS in both sexes. Aortic diameter and aortic wall thickness were unchanged in all groups (Figure 2D,E). To determine whether changes in collagen, elastin, and wall thickness resulted in changes in mechanical properties of the vessel, ultrasound imaging was used to determine pulse wave velocity (PWV): a measure of aortic stiffening. However, PWV in the abdominal aorta remained unchanged (Figure 2F).

### 3.3 Sex steroids increased over time regardless of diet in both sexes

Plasma progesterone was increased from baseline to the end of the diet regime in NCD (*p* = .01; Table 3) and HFSS (*p* = .004; Table 3) in males and only in HFSS female mice (*p =* .04; Table 3). Plasma estradiol was increased from baseline to week 10 in NCD females only (*p* = .02; Table 3).

**TABLE 3:** Plasma progesterone, testosterone, and estradiol in normal control diet (NCD) and high fat salt and sugar (HFSS) diet‐fed male and female mice.

	Progesterone (ng/mL)		Testosterone (ng/mL)		Estradiol (ng/mL)	
	Baseline	Week 10	Baseline	Week 10	Baseline	Week 10
Males						
NCD	22.9 ± 3.0	40.0 ± 3.3*	0.2 ± 0.1	0.1 ± 0.05	N/A	N/A
HFSS	22.2 ± 2.5	49.9 ± 5.6*	0.2 ± 0.1	0.5 ± 0.3	N/A	N/A
Females						
NCD	42.3 ± 7.1	55.69 ± 7.82	N/A	N/A	0.3 ± 0.03	0.3 ± 0.02*
HFSS	50.3 ± 9.6	62.8 ± 4.9*	N/A	N/A	0.3 ± 0.02	0.3 ± 0.03

*Note*: Values are expressed as mean ± SEM, *p < .05 baseline vs. week 10, mixed effects analysis with Tukey's multiple comparisons test.

Abbreviation: N/A, not applicable.

### 3.4 HFSS‐diet increased serum adiponectin at the endpoint in males

Adiponectin is an adipocytokine released by adipose tissue and known to have important protective effects on the vasculature in metabolic syndrome.^44^ Therefore, we assessed circulating adiponectin levels. HFSS diet caused serum adiponectin to increase at the endpoint in males only (compared to baseline; Table 4; *p* = .001). Adiponectin levels remained constant throughout the diet regimen in all other groups (Table 4).

**TABLE 4:** Circulating adiponectin in normal control diet (NCD) and high fat, salt, and sugar (HFSS) diet‐fed male and female mice.

	Baseline	Week 6	Week 10
Males			
NCD	8.91 ± 0.97	7.83 ± 0.88	9.18 ± 0.49*
HFSS	6.63 ± 0.81	9.19 ± 0.69	10.49 ± 0.44
Females			
NCD	13.07 ± 1.26	14.88 ± 1.87	14.22 ± 2.56
HFSS	10.24 ± 0.65	10.12 ± 1.81	13.81 ± 1.22

*Note*: Values are expressed as mean ± SEM, *p < .05 baseline vs. week 10, mixed‐effects analysis with Tukey's multiple comparisons test.

### 3.5 PVAT impairs endothelium‐dependent vasorelaxation in HFSS males and NCD females

**FIGURE 3:**
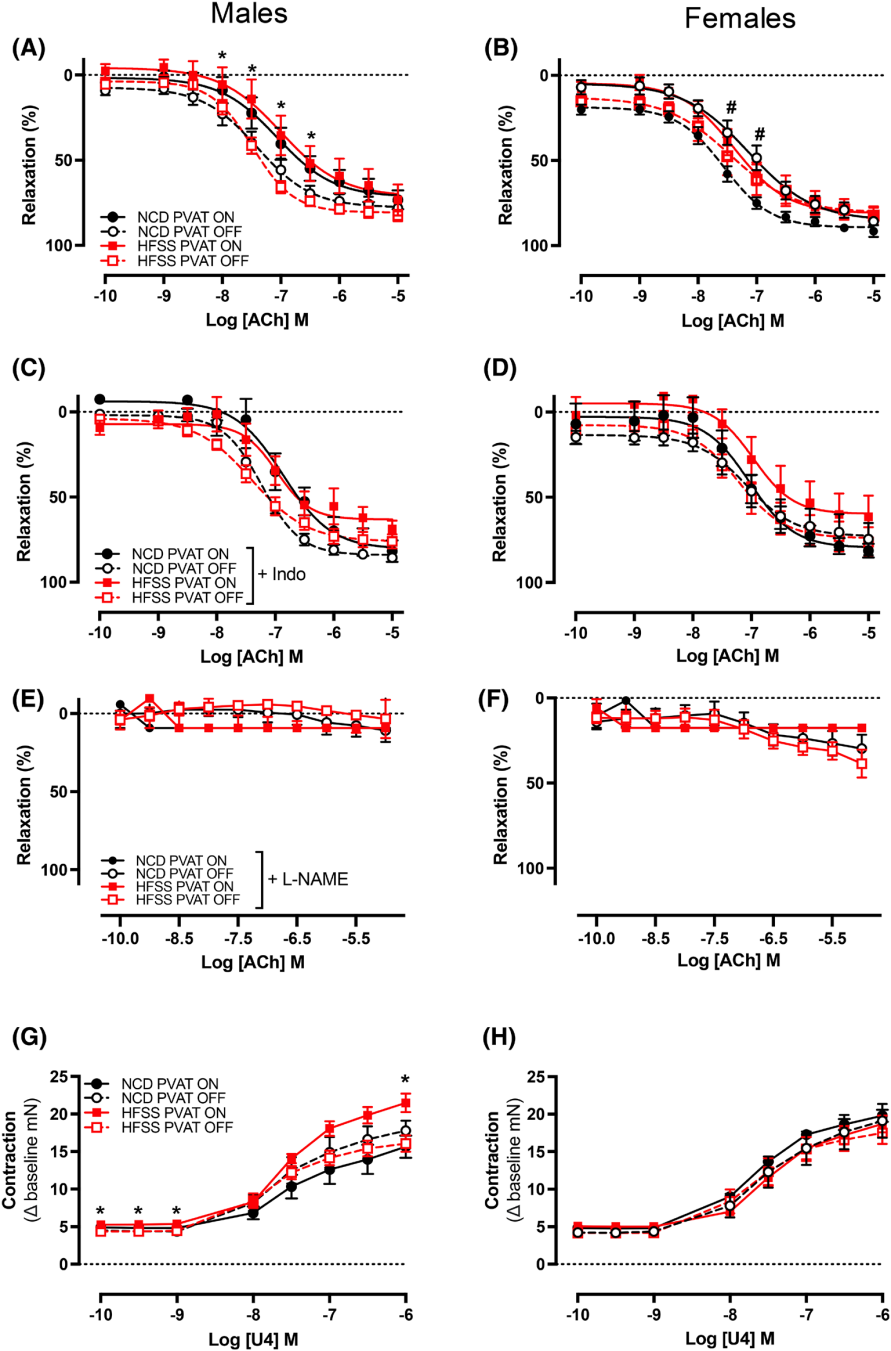
Presence of perivascular adipose tissue impaired endothelium‐dependent relaxation and increased contractions to U4 in high‐fat, sugar, and sugar‐fed male mice and in normal chow females. Concentration‐response curves to acetylcholine (ACh) in the absence (NCD: ○ and HFSS: □) and presence (NCD: ● and HFSS: ■) of perivascular adipose tissue (A and B), indomethacin (C and D), L‐NAME (E and F) and to thromboxane U46619 mimetic (G and H) in C57BL/6 male and female mice fed a normal chow diet or high‐fat, salt, and sugar. A and B *n* = 8–13 per group, C and F *n* = 7–11 per group, G and H *n* = 6–10 per group. Values are expressed as mean ± SEM. *p* < .05 for 2‐way ANOVA with Sidak's multiple comparison and unpaired *t*‐test. *HFSS PVAT on vs. HFSS PVAT off, ^#^NCD PVAT on vs. NCD PVAT off.

In isolated vascular ring experiments, ACh‐response curves showed impaired relaxation in HFSS‐males when PVAT was left intact versus HFSS‐males when PVAT was absent (*p* < .05; Figure 3A). There were no differences in ACh‐mediated relaxation in NCD‐males regardless of whether PVAT was present or absent (Figure 3A). In contrast, endothelium‐dependent relaxation was reduced in NCD‐females when PVAT was left intact versus NCD‐females when PVAT was absent (*p* < .05; Figure 3B). However, the presence and/or absence of PVAT did not affect ACh‐mediated relaxation in HFSS‐females (Figure 3B). To investigate the contribution of endothelium‐derived factors in MetS‐induced endothelial dysfunction, concentration‐response curves to ACh were evaluated in the presence of the NOS inhibitor, L‐NAME (Figure 3C,D) or the COX inhibitor, indomethacin (Figure 3E,F). There were no differences in ACh‐mediated relaxation between diet groups in the presence of either blocker. There were also no differences in maximum relaxation (Table 5; *R*_max_) and sensitivity to ACh (Table 5; pEC_50_) between diet and PVAT groups in male or female mice.

**TABLE 5:** Half‐maximal effective concentration (pEC_50_) and maximal response (%, *R*_max_) to acetylcholine (ACh) and the thromboxane mimetic U46619 in the aorta of male and female mice fed a normal control diet (NCD) and high fat, salt, and sugar (HFSS) diet.

	ACh *R*_max_ (%)	ACh pEC_50_	U46619 *R*_max_ (%)	U46619 pEC_50_
Males				
NCD PVAT on (*n* = 5–9)	90.52 ± 2.03	−7.44 ± 0.35	19.78 ± 0.79	−7.58 ± 0.08
NCD PVAT off (*n* = 6–8)	90.03 ± 3.92	7.40 ± 0.16	19.12 ± 2.26	−7.60 ± 0.18
HFSS PVAT on (*n* = 9–10)	62.27 ± 7.25	−6.78 ± 0.22	21.47 ± 1.25	−7.60 ± 0.05
HFSS PVAT off (*n* = 7–10)	76.47 ± 3.21	−7.53 ± 0.21	16.06 ± 1.09*	−7.80 ± 0.06
Females				
NCD PVAT on (*n* = 6–8)	86.19 ± 1.82	−6.95 ± 0.15	19.78 ± 0.80	−7.67 ± 0.05
NCD PVAT off (*n* = 6–7)	90.45 ± 3.61	−7.56 ± 0.09	19.12 ± 2.26	−7.51 ± 0.09
HFSS PVAT on (*n* = 7–9)	79.29 ± 4.30	−7.22 ± 0.12	18.69 ± 1.11	−7.41 ± 0.12
HFSS PVAT off (*n* = 8–10)	81.28 ± 7.26	−7.25 ± 0.22	17.49 ± 1.46	−7.68 ± 0.12

*Note*: Values are expressed as mean ± SEM, *p < .05 vs. PVAT off, 2‐way ANOVA with Tukey's multiple comparisons test.

### 3.6 PVAT enhances U46619‐mediated contraction in HFSS males only

We assessed contraction responses to thromboxane mimetic U46619. The presence of HFSS‐PVAT in males increased aortic contractility to U46619, whereas PVAT had no influence on the response in NCD males (*p* < .05; Figure 3G). There were no differences in response to U46619 in the females irrespective of diet or the presence or absence of PVAT (Figure 3H). Maximum contraction from baseline was greater in HFSS males when PVAT was left intact (*p =* .005; Table 5) with no differences in females (Table 5) and no changes in sensitivity to U46619 (Table 5; pEC_50_) for both sexes.

### 3.7 HFSS increased the expression of immune cell regulation genes in female PVAT and ion transport genes in male PVAT

**FIGURE 4:**
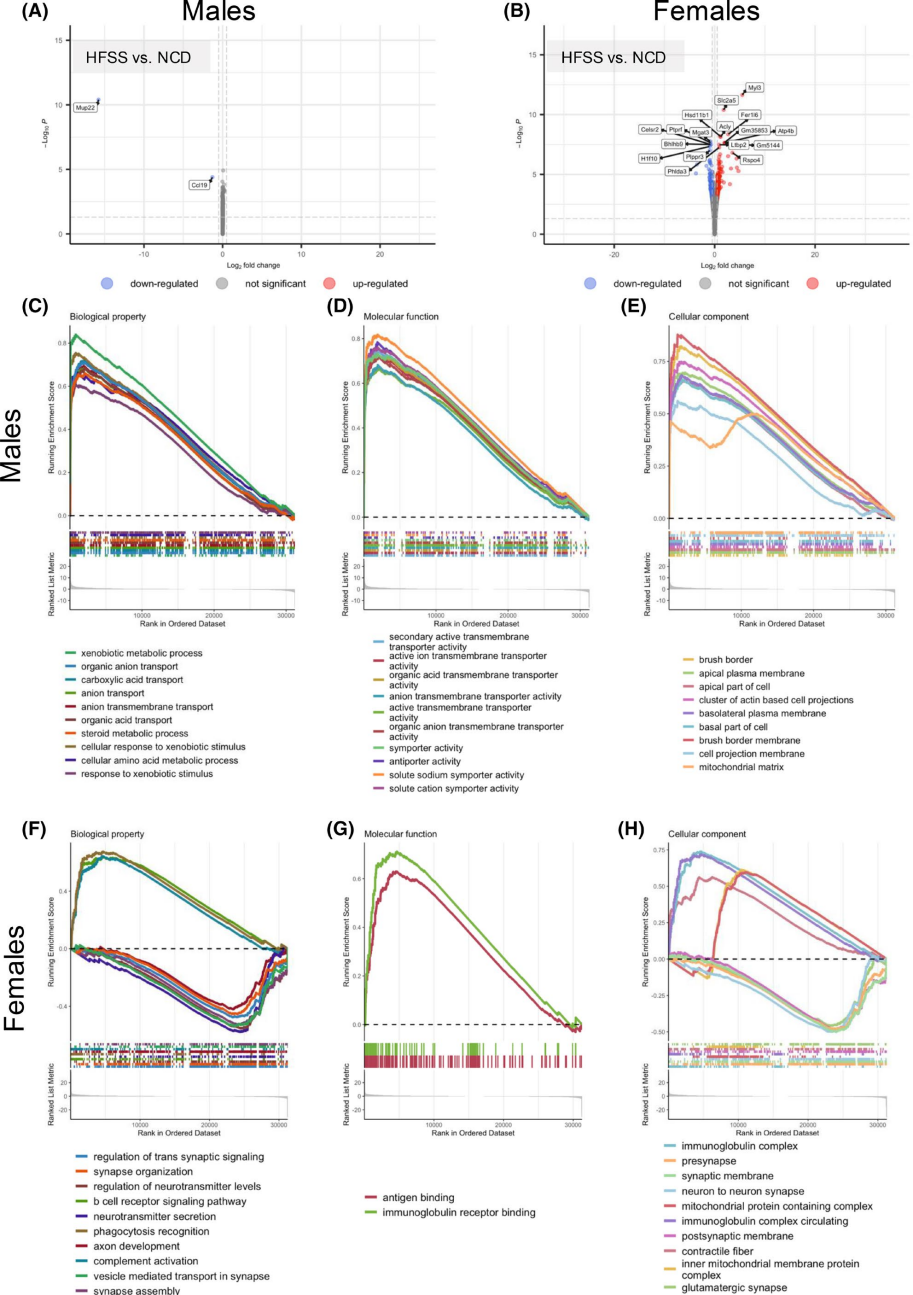
Top 10 significantly altered genes and gene sets in HFSS and NCD PVAT male and female mice. Volcano plots showing pair‐wise comparisons of differentially expressed genes (A: male PVAT; B: female PVAT). Graphical representation of gene set enrichment analysis (GSEA) of biological property (C: males; F: female), molecular function (D: male; G: female) and cellular component (E: male; H: female). Positive Running Enrichment Scores indicated an upregulation of gene sets in the HFSS group, whereas negative Running Enrichment Scores indicated upregulation in NCD gene sets. Each vertical dash represents a gene list ordered by decreasing log2‐fold values.

Having identified sex‐specific functional roles of PVAT in the aorta, we sought to further investigate the changes in gene expression in the PVAT in response to a HFSS diet using bulk‐RNA‐seq. Volcano plots showed pairwise comparisons between differentially expressed genes (Figure 4A,B) and GSEA showed increased biological properties, molecular functions, and cellular components associated with HFSS PVAT. GSEA of PVAT in HFSS males showed increases in biological properties, molecular functions, and cellular components involved in *organic anion transport*, *carboxylic acid transport*, *arachidonic acid metabolic processes*, *sodium ion transport*, *brush border*, and *mitochondrial matrix* (Figure 4C–E). GSEA showed increased biological properties, molecular functions, and cellular components associated with immune responses in the HFSS‐PVAT in females. This included *B cell receptor signaling pathway*, *complement activation*, *antigen binding, immunoglobulin receptor binding*, and *immunoglobulin complex* (Figure 4F–H). Biological properties and cellular components involved in neurotransmitter secretion were decreased in HFSS‐PVAT compared to NCD‐PVAT in female mice (Figure 4F). Specifically, gene sets associated with *synaptic synapse assembly*, *vesicle‐mediated transport in synapse*, *postsynaptic membrane*, *and glutamatergic synapse* (Figure 4F,H).

### 3.8 HFSS had no effect on aortic leukocytes and lymphocytes in males and females

**FIGURE 5:**
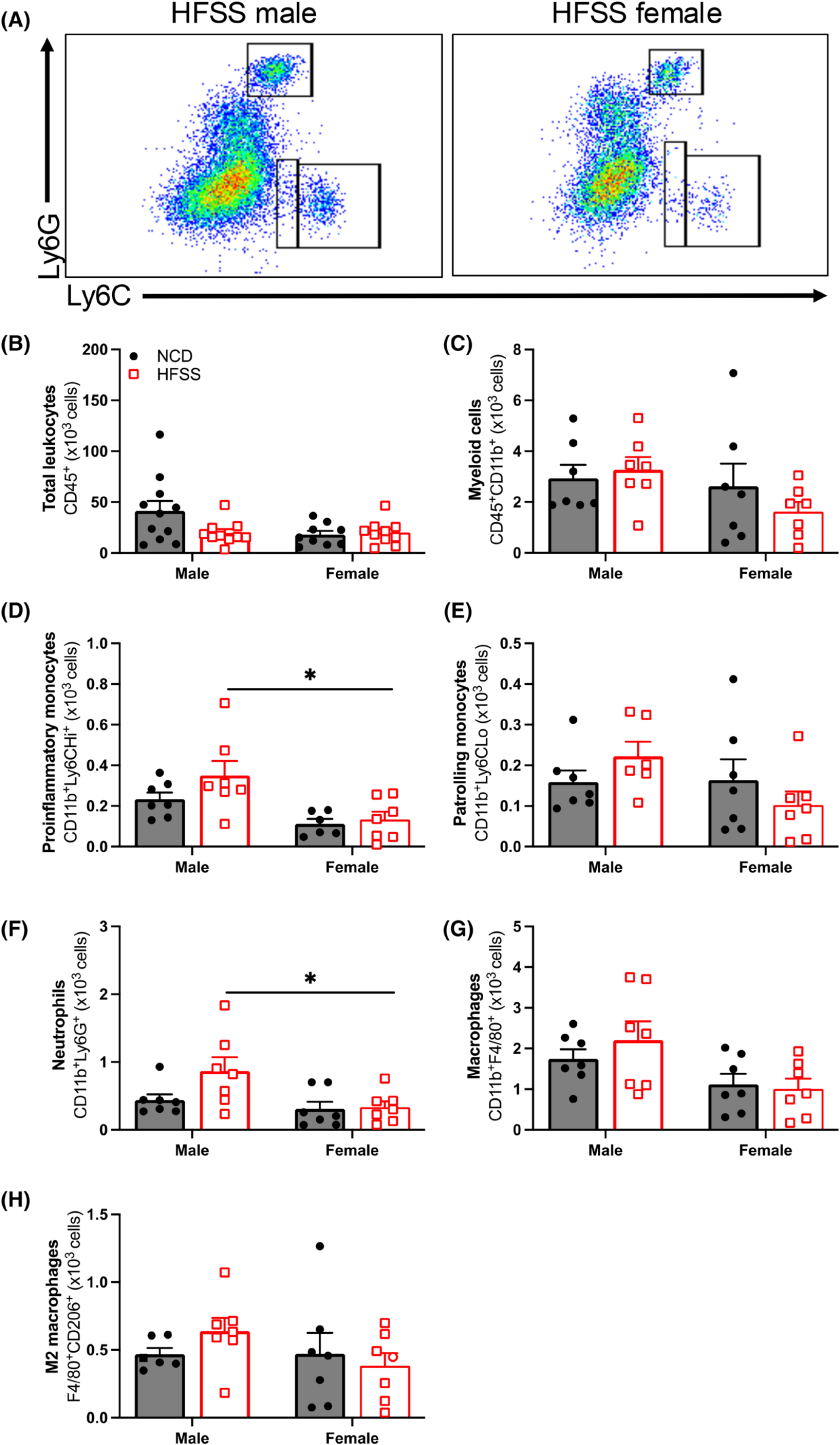
High‐fat, salt, and sugar diet did not affect aortic leukocyte populations; however, a sex difference was observed. Representative aortic flow cytometry plots (A), total leukocytes (CD45^+^; B), myeloid‐derived cells (CD11b^+^; C), proinflammatory monocytes (Ly6C^Hi^; D), patrolling monocytes (Ly6C^Lo^; E), neutrophils (Ly6G^+^; F), macrophages (F4/80^+^; G), and M2 macrophages (CD206^+^; H) in the aortas from C57BL/6 male (*n* = 5–8 per group) and female (*n* = 7–11 per group) mice fed a normal chow diet (NCD; black; ●) or high‐fat, salt, and sugar diet (HFSS; red; □). Values are expressed as mean ± SEM. *p < .05 HFSS males vs. HFSS females for 2‐way ANOVA with Tukey's multiple comparison.

**FIGURE 6:**
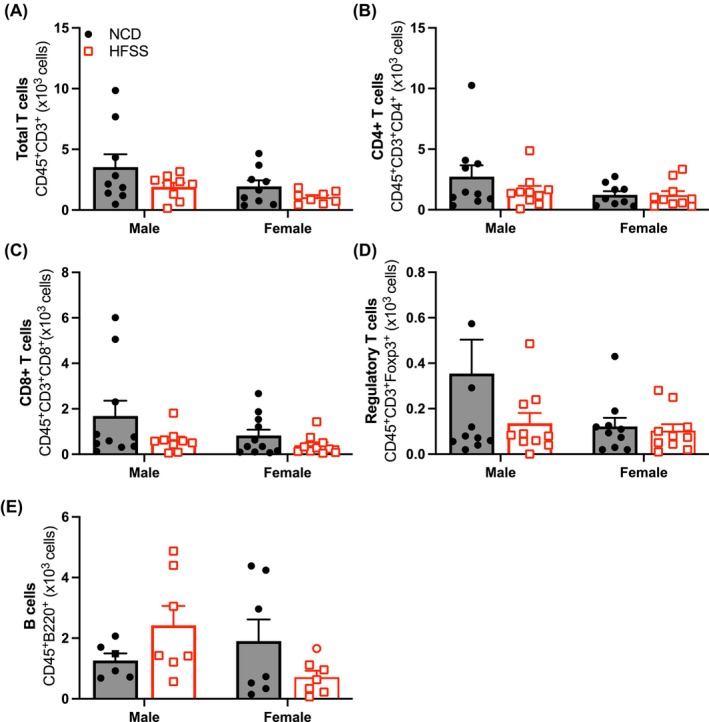
High‐fat, salt, and sugar diet did not affect aortic lymphocyte populations. Total T cells (CD3^+^; A), T helper/Treg cells (CD4^+^; B), cytotoxic T cells (CD8^+^; C), double‐negative T cells (CD4^−^CD8^−^; D) and B cells (B220^+^; E) in the aortas from C57BL/6 male and female mice fed a normal chow diet (NCD; black; ●) or high‐fat, salt, and sugar diet (HFSS; red; □). Values are expressed as mean ± SEM, *n* = 7–11 per group.

Flow cytometry revealed that HFSS had no effect on the total number of immune cells or any of the subsets studied in the aorta and PVAT (Figures 5 and 6). However, proinflammatory monocytes (CD11b^+^Ly6Hi^+^) and neutrophils (CD11b^+^Ly6G^+^) were higher in HFSS males compared to HFSS females, regardless of diet (*p* < .05; Figure 5D,F).

## 4 DISCUSSION

The differences in vascular responses to MetS between sexes and rodent species are still poorly understood. There is a bias toward studying males due to their increased susceptibility to developing MetS. Our study addressed this gap by characterizing vascular and PVAT function in a novel mouse model inducing MetS in both sexes. We showed that a 10‐week HFSS diet induced all five of the metabolic disturbances in male and female mice. Notably, PVAT impaired aortic endothelial function in HFSS‐fed males and in NCD females. This dysfunction was linked with increased aortic collagen deposition and perivascular adipocyte hypertrophy, with no impact on aortic immune cells in both sexes. Bulk RNA sequencing revealed that HFSS induced sex‐specific changes to PVAT function with increased expression of ion transport genes in males and increased expression of immune cell regulation genes in females. These findings highlight sex differences in vascular and PVAT function and sex‐specific responses in MetS.

HFSS diet increased the rate of cumulative weight gain, which was associated with aortic perivascular adipocyte hypertrophy in both sexes. This correlates with other models of obesity, where perivascular adipocyte hypertrophy occurs to accommodate the increased demand for fat storage due to increased caloric intake.^45^ This adipocyte hypertrophy is often associated with leukocyte infiltration; however, this was not observed in our study in either sex. Our study also showed aortic fibrosis in HFSS‐fed mice; however, no changes in aortic elastin content.^46^ This fibrosis is likely acute remodeling of the extracellular matrix to facilitate the growth of hypertrophic adipocytes in the PVAT.^47^, ^48^ Despite the increases in aortic adiposity and fibrosis, there were no differences in pulse wave velocity (PWV) in the abdominal aorta for either sex. Arterial stiffness, typically measured by PWV, is associated with hypertension and metabolic risk factors, reflecting the loss of arterial wall compliance to expansion with increasing volume.^49^, ^50^ However, PWV is a measure of passive function, and while increases in collagen were observed, it did not result in detectable changes in PWV and passive blood flow. Considering that the HFSS diet only caused transient increases in SBP and hyperglycemia toward the end of the diet regime, the pathological remodeling was likely not advanced enough to cause changes in vascular stiffness.

Circulating levels of glucose, lipids, and adiponectin are markers of metabolic dysregulation and clinical risk factors for cardiometabolic disease.^51^ Adiponectin, an insulin‐sensitizing hormone, is secreted by adipocytes and exhibits an inverse relationship with adiposity, with lower levels associated with conditions such as type 2 diabetes, hypertension, and MetS.^52^, ^53^, ^54^, ^55^ This contrasts with our study, where adiponectin levels were increased in HFSS‐fed males and unchanged in HFSS‐fed females. Our findings are also at odds with previous studies in humans and rodents that report higher circulating adiponectin levels in females compared to males.^56^, ^57^ Due to the relatively short duration of the HFSS diet regime, the increased adiponectin in males may be an early compensatory response.

Given the focus on sex‐specific effects, circulating sex steroids, particularly plasma progesterone, were seen to increase in all animals over time. Progesterone, as a gonadal hormone, affects lipid, carbohydrate, and protein metabolism and promotes body fat deposition.^58^ As the mice are aging over the 10‐week diet regime, the continued increase in growth and weight gain likely contributes to increased progesterone levels.^59^ Moreover, physiological stress and the estrus cycle can also contribute to increased levels of progesterone; however, we have not explored this in our study.^60^ Plasma estradiol was increased in NCD females over time. The Barostoc feed used for NCD may contain isoflavones, a natural estrogenic compound found in legumes.^61^ This effect was not observed in the HFSS‐female group, suggesting a loss of estradiol's protective cardiometabolic effects, which are well documented.^62^ A limitation of our study is the absence of estradiol and testosterone assays in both sexes due to limited sample size. Additionally, we did not account for the estrus cycle in female mice, which studies suggest can influence blood pressure, blood glucose, and estradiol levels.^63^ By not including this, we may have overlooked potential differences between our experimental groups and not highlighted possible differences in males. Furthermore, we are also aware of the challenges associated with measuring plasma steroid levels using direct immunoassays, especially at low concentrations.^64^, ^65^ However, the assays were sensitive enough to detect estradiol and testosterone levels in our study.

Our flow cytometry data showed no effect of HFSS on aortic immune cells in either sex. While this is consistent with findings from a previous study in male and female mice fed a high‐fat diet for 10 weeks,^19^ it contrasts with other studies characterizing leukocytes in visceral adipose tissues. Specifically, CD8^+^ T cells were increased and CD4^+^ and regulatory T cells decreased in the visceral adipose of obese males when compared to females and their NCD counterparts.^21^, ^66^, ^67^ Interestingly, Ly6Chi^+^ proinflammatory monocytes and Ly6G^+^ neutrophils were increased in aortas from HFSS males compared to HFSS females, suggesting a sex difference in immune cell composition. This data also contradicts findings from other rodent studies in other adipose beds. In healthy C57BL/6 mice, the total number of macrophages in females was approximately twice that in males.^21^ The discrepancies between studies may be explained due to the different adipose tissue beds being studied. Moreover, the studies that showed leukocyte infiltration in adipose tissue used longer diet regimes. Further research is required to determine whether leukocyte infiltration occurs in PVAT in prolonged diet regimes.

Endothelial dysfunction is commonly observed in each of the metabolic abnormalities of MetS.^68^ For this study, we also considered the role of PVAT in MetS. We observed that PVAT blunted endothelium‐dependent relaxation and enhanced U46619‐mediated contraction in HFSS males. The presence of PVAT also blunted endothelium‐dependent relaxation in NCD females. These results indicate a male‐specific mechanism in response to HFSS that alters vascular tone through the modulation of PVAT function. Our findings correlate with other rodent models of HFD‐induced obesity, hypertension, and hyperglycemia, where the presence of PVAT reduces endothelium‐dependent aortic relaxation and increases contractility.^4^, ^69^, ^70^ In these earlier studies, vascular dysfunction was shown to occur through various mechanisms, including reduced NO bioavailability, altered release of adipocyte‐derived relaxing and contractile factors, and vascular smooth muscle cell proliferation.^4^, ^69^, ^70^ These studies only reported findings in males and, to our knowledge, our study is the first to explore the role of PVAT in females in the setting of MetS. It is possible that the HFSS diet regime did not alter vascular function in females because the metabolic disturbances observed were not as severe as in males. One limitation of the pin myography experiments was the use of only one vasoconstrictor — U46619. While the use of other vasoconstrictors (such as phenylephrine) may have provided insights into thromboxane‐independent constriction, U46619 is most used in mouse aortas due to its ability to induce consistent and long‐lasting constriction. It is also unclear whether the anti‐vasodilatory effect of PVAT in the aortas of NCD females is beneficial.

RNA sequencing data provided further information regarding the function of PVAT in NCD females. Indeed, gene sets relating to neurotransmitter secretion and transport, as well as vesicle‐mediated transport in synapses, were enriched in the PVAT from NCD females. This suggests that PVAT influences aortic function in a contractile manner via the sympathetic nervous system. This observation aligns with our pin myography data, where the presence of PVAT blunted ACh‐mediated relaxation in NCD females but had no effect on U46619‐mediated vasoconstriction. It is possible that the effect of the PVAT exerts a subtle anti‐relaxant effect in NCD females that restricts ACh‐mediated relaxation, without augmenting contractile properties through the thromboxane mimetic pathway. Moreover, PVAT adipokines such as leptin, resistin, and TNF‐α can reduce adiponectin production, in turn affecting NO bioavailability.^23^

HFSS caused upregulation of genes in the female aortic PVAT related to increased fatty acid synthesis and storage (*Acly*), fructose transporter due to increased fructose in the diet (*Slc2a5*), and inflammation and oxidative stress (*Fer1l6* and *Ltbp2*). In particular, *Ltbp2* is associated with extracellular matrix proteins that are regulators of transforming growth factor beta.^71^ Therefore, in response to the HFSS diet, *Ltbp2* upregulation could be due to PVAT remodeling observed in our histology data. In contrast, downregulated genes were related to the disruption of lipid metabolism and insulin signaling (*Mgat3*, *Plppr3* and *Ptprf*). Excess dietary lipids from the HFSS can impair lipid metabolism by decreasing lipid uptake, synthesis, and storage, possibly also serving as an adaptive response. Such that *Mgat2*‐deficient mice show increased energy expenditure and protection from metabolic disorders,^72^ highlighting that downregulation of these genes may act as a protective mechanism.

Bulk RNA sequencing also revealed insights into the biological processes in aortic PVAT in HFSS females, where the top upregulated pathways were related to immune cell regulation, particularly B cell activity. The bulk RNA sequencing was performed on the abdominal aortic PVAT, which is predominately white adipose tissue. In metabolic diseases such as obesity, white adipose tissue expansion is linked to local inflammation from immune cell infiltration, fibrosis, and disrupted mitochondrial function.^73^, ^74^, ^75^ Moreover, while our data show that total aortic B cell numbers were not altered by HFSS, our bulk RNA sequencing data suggest that B cell activity may have been increased in HFSS females. Due to limitations to the size of the antibody panel, we were unable to quantify specific B cell subsets in this study.

Compared to females, there were striking differences in differentially expressed genes in HFSS‐male PVAT. Compared to NCD, we observed downregulation of *Mup22* and *Ccl19*. MUP is a family of major urinary proteins highly expressed in the liver. MUP expression is sexually dimorphic, with higher expression in males than in females, regulated by hormones such as testosterone and growth hormones.^76^ MUP expression is also associated with metabolism, where lower MUP concentrations in plasma are found in obese and diabetic mice.^77^, ^78^ This suggests that downregulation of *Mup22* in PVAT is due to metabolic disturbances linked to hormonal changes. While *Ccl19* expression is found to be significantly higher in obese individuals compared with lean individuals,^79^ our study found it to be downregulated in response to a HFSS diet. This difference may be due to other studies focusing on subcutaneous adipose tissue, whereas aortic PVAT is a type of visceral adipose tissue and has a different inflammatory profile.

Biological processes that were enriched in the PVAT of HFSS males compared to NCD males were related to xenobiotic metabolic processes and ion transport, including organic anion transport, carboxylic acid transport, and sodium ion transport Many diet‐derived compounds such as polyphenols, phytoestrogens, lipids, and preservatives can be classified as xenobiotics.^80^ These exogenous substances, which can promote systemic inflammation, stimulate the expression of xenobiotic receptors.^80^ Moreover, xenobiotics can directly act as agonists/antagonists of hormone signaling or indirectly alter hormone metabolism rates and circulating hormone levels.^81^ Modulation of P450 expression by xenobiotics can affect the metabolism of foreign chemicals and steroid hormones.^81^ Xenobiotic metabolism can also influence endogenous metabolism. Although not extensively explored, the host metabolome comprises endogenous chemicals involved in processes such as glycolysis, the citric acid cycle, and the fatty acid β‐oxidation pathway.^80^ Additionally, pathways related to active ion transporter activity and organic anion transport were regulated. This gene set is associated with sodium‐dependent organic anion transporters, facilitating the transport of sulfated steroid hormones like estrone sulfate and dehydroepiandrosterone sulfate into target cells.^82^ This suggests sex‐specific responses to hormones that influence vascular function, and in this study may modulate NO production to reduce dilation.^83^

Our study is the first comprehensive characterization of sex‐specific effects in response to a HFSS diet, including metabolic parameters, vascular inflammation, and vascular function. HFSS induced metabolic abnormalities in both sexes, but males were more susceptible to these effects. Vascular dysfunction, particularly endothelial dysfunction and increased contractility, occurred in the presence of PVAT in HFSS males. Interestingly, NCD females exhibited impaired endothelium‐dependent relaxation in the presence of PVAT. This highlights major sexual dimorphisms in aortic PVAT function in both healthy and diseased conditions. Furthermore, HFSS induced aortic collagen deposition and increased adipocyte size in both sexes. HFSS did not impact aortic immune cell infiltration, but a sex effect on immune cells was evident in HFSS‐fed mice. Our findings highlight distinct mechanisms underlying MetS development in males and females in a new clinically relevant model of MetS, emphasizing the differing roles of PVAT in the two sexes. Further research is required to uncover the specific mechanisms that lead to these sex differences in MetS. The new HFSS mouse model we developed in our study could serve as a powerful new tool to facilitate this research and identify new targets for sex‐specific therapies to reduce vascular dysfunction in MetS.

## AUTHOR CONTRIBUTIONS

V. Tran, G. R. Drummond, A. Vinh, and M. Jelinic conceived and designed the research. V. Tran, H. Brettle, H. Diep, H. B. F. Galvao, A. Vinh, and M. Jelinic performed the research and acquired the data; V. Tran, H. Brettle, H. B. F. Galvao, C. G. Sobey, G. R. Drummond, A. Vinh, and M. Jelinic analyzed and interpreted the data. All authors were involved in drafting and revising the manuscript.

## DISCLOSURES

The authors declare no conflicts of interest.

## Supporting information


Appendix S1.

## Data Availability

The data that support the findings of this study are available in the Materials and Methods, Results, and/or Supplemental Material of this article.
