# Supplementary material for: Sex‐specific characterization of aortic function and inflammation in a new diet‐induced mouse model of metabolic syndrome
Source: FASEB J. 2025 Mar 4;39(5):e70413. doi: 10.1096/fj.202401871R (PMC11878204; doi:10.1096/fj.202401871R)
Supplement: Supplementary file 1 — Appendix S1. [file FSB2-39-e70413-s001.pdf]

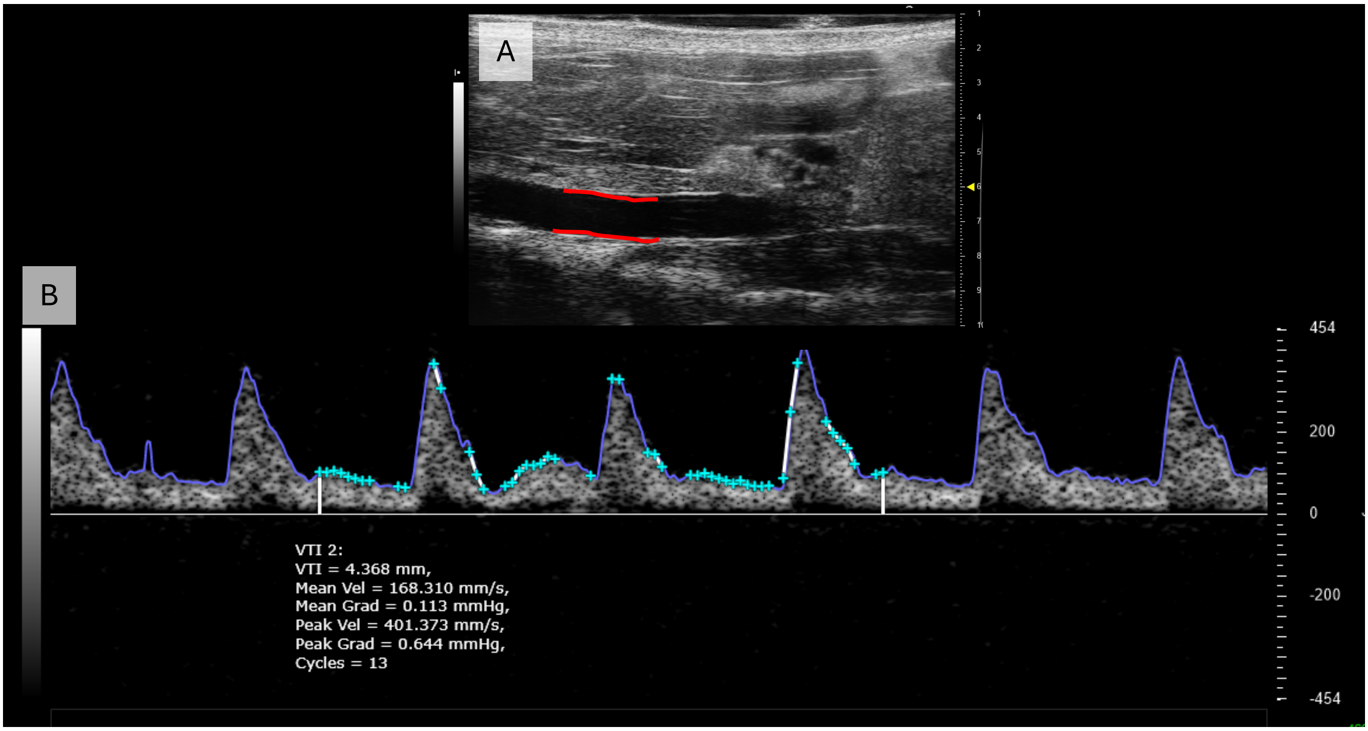

Supp Fig 1. Representative of pulse-wave velocity measurement.
B-mode image using edge detection and contour tracking technique of abdominal aorta **(A)**. PW-Doppler images are processed for the identification of the envelope signal from which the single-beat mean velocity waveform is obtained **(B)**.

Supp Table 1: Mouse antibodies used for flow cytometry. All antibodies were purchased from Biolegend and eBioscience (San Diego, California, USA)

Antibody	Clone	Fluorophore	Concentration	Catalogue #
CD45	30-F11	AF700	1 mg/ml	103128
CD11b	M1/70	BV421	0.5 mg/ml	101236
Ly6C	HK1.4	FITC	0.2 mg/ml	128005
Ly6G	1A8	PE-Cy7	0.2 mg/ml	127618
F4/80	BM8	APC-Cy7	0.4 mg/ml	123118
CD3e	17A2	APC	0.4 mg/ml	100236
CD4	RM4-5	BV605	0.4 mg/ml	100548
CD8a	53-6.7	PerCP Cy5.5	0.2 mg/ml	100734
B220	RA3-6B2	PE	0.2 mg/ml	103208
Foxp3	FJK-16s	PE-Cy5.5	0.4 mg/ml	35577382
CD206	C068C2	PE-DAZZLE	0.4 mg/ml	141731

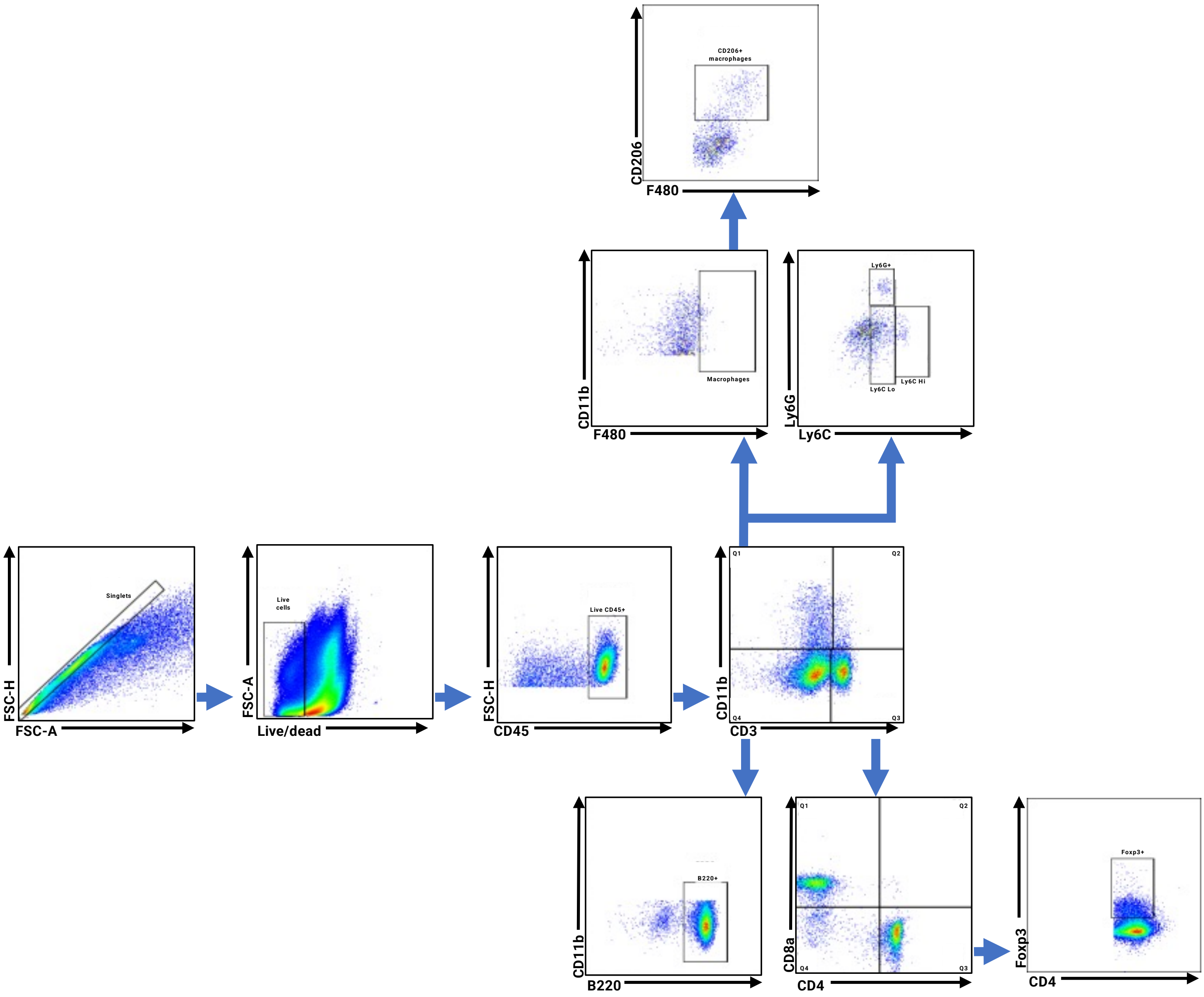

Supp Fig 2. Gating strategy for flow cytometric analysis.
Leukocytes were gated as $CD45^+$ populations against forward scatter (FSC). Total leukocytes, i.e. live leukocyte singlets, were gated by FSC -height vs FSC-area, and exclusion of dead cells (live/dead stain). Total leukocytes were then divided into myeloid cells ($CD11b^+$) and T cells ($CD3^+$). Some of the myeloid cells were identified as macrophages ($CD11b^+F4/80^+$), M2 macrophages ($F4/80^+/CD206^+$) proinflammatory monocytes (Ly6CHi), patrolling monocytes (Ly6CLo) and neutrophils ($Ly6G^+$). B cells were positive for $B220^+$. T cells that were positive for CD3 staining were classified as $CD4^+$, or $CD8^+$ T cells. $CD4^+$ T cells were further classified to $Foxp3^+$ T cells.
